# Cold-inducible RNA-binding protein (CIRP) causes sepsis-associated acute lung injury via induction of endoplasmic reticulum stress

**DOI:** 10.1038/srep41363

**Published:** 2017-01-27

**Authors:** Mohammad Moshahid Khan, Weng-Lang Yang, Max Brenner, Alexandra Cerutti Bolognese, Ping Wang

**Affiliations:** 1 Center for Immunology and Inflammation, The Feinstein Institute for Medical Research, Manhasset, NY 11030, USA; 2 Department of Surgery, Hofstra Northwell School of Medicine, Manhasset, NY 11030, USA; 3 Elmezzi Graduate School of Molecular Medicine, Manhasset, NY 11030, USA

## Abstract

Cold-inducible RNA-binding protein (CIRP), released into the circulation during sepsis, causes lung injury via an as yet unknown mechanism. Since endoplasmic reticulum (ER) stress is associated with acute lung injury (ALI), we hypothesized that CIRP causes ALI via induction of ER stress. To test this hypothesis, we studied the lungs of wild-type (WT) and CIRP knockout (KO) mice at 20 h after induction of sepsis by cecal ligation and puncture (CLP). WT mice had significantly more severe ALI than CIRP KO mice. Lung ER stress markers (BiP, pIRE1α, sXBP1, CHOP, cleaved caspase-12) were increased in septic WT mice, but not in septic CIRP KO mice. Effector pathways downstream from ER stress – apoptosis, NF-κB (p65), proinflammatory cytokines (IL-6, IL-1β), neutrophil chemoattractants (MIP-2, KC), neutrophil infiltration (MPO activity), lipid peroxidation (4-HNE), and nitric oxide (iNOS) – were significantly increased in WT mice, but only mildly elevated in CIRP KO mice. ER stress markers were increased in the lungs of healthy WT mice treated with recombinant murine CIRP, but not in the lungs of TLR4 KO mice. This suggests CIRP directly induces ER stress via TLR4 activation. In summary, CIRP induces lung ER stress and downstream responses to cause sepsis-associated ALI.

Sepsis is defined as life-threatening organ dysfunction caused by a dysregulated host response to infection and results in an estimated 5.3 million deaths worldwide every year^1^,^2^. Sepsis is often complicated by respiratory dysfunction due to acute lung injury (ALI), which is characterized by acute hypoxemic respiratory failure with bilateral pulmonary infiltrates and has an independent mortality rate of over 38% 3. Hematogenic ALI is generally thought to develop as a consequence of leukocyte and pulmonary endothelial cell activation by microbial products or cell injury-associated endogenous molecules^4^. However, the precise mechanisms underlying cellular activation and leading to ALI are still poorly understood, limiting the discovery of effective treatments for this condition.

Cold-inducible RNA-binding protein (CIRP) is a highly conserved nuclear protein whose gene expression is upregulated by hypoxia and mild hypothermia^5^,^6^. We have discovered that, during sepsis, CIRP translocates from the nucleus to the cytoplasm and is released into the circulation^7^. Once in the circulation, CIRP acts as a damage-associated molecular pattern molecule (DAMP) by binding to the TLR4-MD2 receptor complex to increase sepsis severity and mortality rate^7^,^8^. We have recently shown that exogenous CIRP administered to healthy mice causes lung injury, as evidenced by vascular leakage, neutrophil infiltration, local production of TNF-α and IL-1β, and activation of the NRLP3 inflammasome in lung vascular endothelial cells^9^. These observations suggest that CIRP plays a critical role in the development of sepsis-associated ALI. The precise mechanism by which CIRP causes lung injury, however, remains to be determined.

Protein folding and assembly normally take place in the endoplasmic reticulum (ER)^10^. Unfolded or misfolded proteins are sensed by the ER transmembrane sentinel proteins IRE1α, PERK, and ATF6, triggering integrated signaling pathways that lead to the unfolded protein response (UPR)^10^,^11^,^12^,^13^. The UPR consists of transcriptional and translational reprogramming that increase the expression of folding and chaperone proteins, cause cell cycle arrest, and down-regulate overall gene expression and protein synthesis^11^,^14^. If the changes in protein expression and chaperone proteins are unable to resolve the ER stress, apoptotic cascades are activated^11^,^14^. The IRE1α pathway, in particular, integrates ER-stress signaling with the inflammatory response. Upon activation, IRE1α homodimerizes, trans-autophosphorylates, and gives rise to spliced XBP1 (sXBP1) through endoribonuclease activity^11^. sXBP1 is a potent transcription factor and a critical regulator of the UPR^15^. Once phosphorylated, the cytoplasmic domain of IRE1α can recruit TRAF2 to activate JNK and IKK, leading to nuclear translocation of AP1 and NF-κB, and to the transcription of numerous genes involved in the inflammatory response^16^,^17^.

ER stress has been observed in sepsis and ALI^10^,^18^,^19^,^20^,^21^,^22^,^23^. Circulating endotoxin, commonly present in sepsis and sepsis-associated ALI, can induce ER stress via TLR4^12^,^19^,^24^,^25^. ER stress then induces the release of proinflammatory cytokines via inflammasome activation^13^,^26^. Additionally, ER stress-induced apoptosis has been implicated in sepsis-associated lymphopenia and liver and myocardial dysfunction, three prominent features of sepsis^12^,^24^,^25^. Therefore, we hypothesized that CIRP released during sepsis leads to sepsis-associated ALI through the induction of ER stress and its downstream events in the lungs.

In this study, we demonstrate that CIRP triggers ER stress and augments inflammation, apoptosis, and histological injury in the lungs. Overall, these findings suggest that CIRP released during sepsis causes and increases the severity of ALI via induction of ER stress.

## Results

### CIRP increases sepsis severity

**Figure 1:**
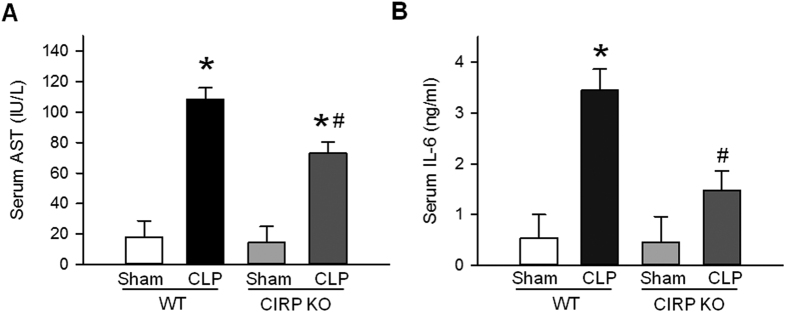
CIRP contributes to systemic injury in sepsis. At 20 h after CLP, serum levels of (**A**) AST (colorimetry) and (**B**) IL-6 (ELISA) were elevated in septic WT mice (*P < 0.05 vs. sham WT mice) and significantly reduced in septic CIRP KO mice (^#^*P* < 0.05 vs. septic WT mice). *Data are expressed as last square mean* ± *SEM. Sample sizes: AST, sham WT* = *4, CLP WT* = *8, sham CIRP KO* = *4, CLP CIRP KO* = *8; IL-6, WT* = *5, CLP WT* = *6, sham CIRP KO* = *4, CLP CIRP KO* = *7*.

To determine the effects of CIRP on sepsis severity, we subjected WT and CIRP KO mice to CLP or sham operation and measured the serum levels of aspartate aminotransferase (AST) and IL-6, two biomarkers of injury and inflammation in sepsis and sepsis-associated ALI^27^,^28^. At 20 h after CLP, the serum levels of AST were 6.0-fold higher in septic WT mice than in sham WT mice (Fig. 1A). In septic CIRP KO mice, however, the serum levels of AST were 35.3% lower than those of septic WT mice (Fig. 1A). Likewise, serum levels of IL-6 were 6.3-fold higher in septic WT mice than in sham WT mice (Fig. 1B). In septic CIRP KO mice, however, the serum levels of IL-6 were 64.7% lower than those of septic WT mice (Fig. 1B). Serum levels of AST and IL-6 in sham CIRP KO mice were similar to those of sham WT mice. These results indicate that CIRP is a significant contributor to systemic injury in sepsis.

### CIRP is associated with increased severity of ALI

**Figure 2:**
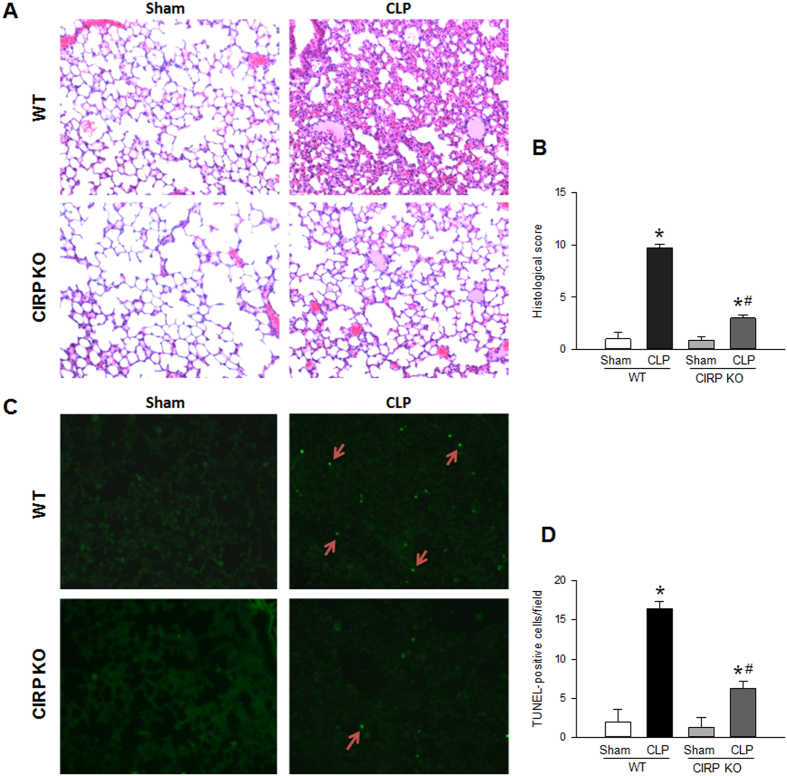
CIRP is associated with histological injury and increases lung apoptosis in ALI. (**A**) The lungs of WT and CIRP KO mice were collected at 20 h after CLP and examined histologically (H&E). Compared with sham WT mice, septic WT mice developed severe lung injury, with septal thickening, hyaline membranes, proteinaceous exudates, microthrombi, and neutrophilic infiltration. Sham CIRP KO mice had normal lung architecture. Septic CIRP KO mice had some proteinaceous exudates and mild neutrophilic infiltration. (**B**) The lung injury histological score was elevated in septic WT mice (*P < 0.05 vs. sham WT mice) and was significantly reduced in septic CIRP KO mice (^#^*P* < 0.05 vs. septic WT mice). (**C**) The lungs of WT and CIRP KO mice were collected at 20 h after CLP and subjected to the TUNEL assay. Compared with sham WT mice, the lungs of septic WT mice had a significant increase in the number of apoptotic events. The lungs of sham CIRP KO mice had no apoptosis. The lungs of septic CIRP KO mice had significantly less apoptotic events than those of septic WT mice. (**D**) The quantity of TUNEL-positive cells was elevated in septic WT mice (*P < 0.05 vs. sham WT mice) and was significantly reduced in septic CIRP KO mice (^#^*P* < 0.05 vs. septic WT mice). *Representative sections; original magnification, 200X. Data are expressed as last square mean* ± *SEM. Sample sizes: histological score, sham WT* = *3, CLP WT* = *12, sham CIRP KO* = *7, CLP CIRP KO* = *12; TUNEL staining, WT* = *3, CLP WT* = *14, sham CIRP KO* = *7, CLP CIRP KO* = *13*.

Lung tissues collected at 20 h after CLP were stained with hematoxylin and eosin (H&E) for histological examination. Septic WT mice developed severe lung injury, with the presence of septal thickening, hyaline membranes, proteinaceous exudates and microthrombi, and the accumulation of neutrophils in the interstitium and alveolar spaces (Fig. 2A,B). Septic CIRP KO mice, however, had mostly preserved lung architecture, and their lung injury histological score was decreased by 75.5% compared to that of septic WT mice (Fig. 2A,B). These results show that CIRP is associated with ALI, demonstrated by increased histological injury in the lungs of septic mice.

### CIRP is associated with apoptosis in ALI

In sepsis, the frequency of pulmonary microvascular endothelial cell apoptosis is increased and is thought to contribute to the endothelial cell dysfunction of ALI^29^,^30^. To determine whether CIRP contributes to apoptosis during ALI, we quantified the number of apoptotic events in the lungs of WT and CIRP KO mice collected 20 h after CLP. As expected, septic WT mice had significantly more apoptotic events than sham WT mice (by 8.2-fold; Fig. 2C,D). The number of apoptotic events in the lungs of septic CIRP KO mice, however, was reduced by 65.2% compared with that of septic WT mice (Fig. 2C,D). There was no significant difference in the number of TUNEL-positive cells in sham WT and CIRP KO mice. These findings indicate that CIRP promotes apoptosis in the lungs of mice with sepsis-associated ALI.

### CIRP increases ER stress in sepsis-associated ALI

**Figure 3:**
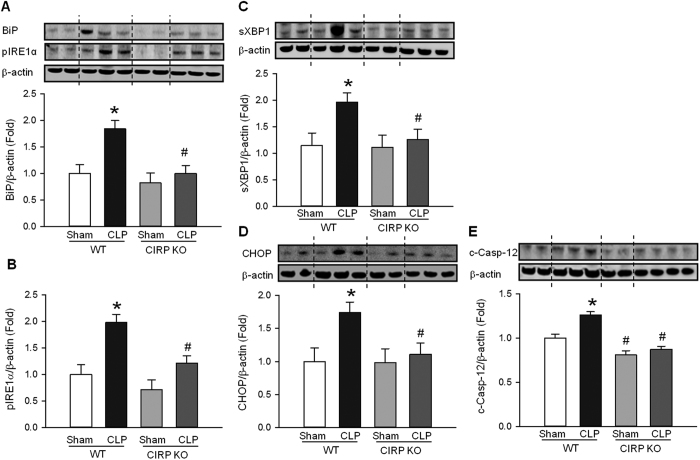
CIRP promotes ER stress during ALI. Lung protein levels of (**A**) BiP, (**B**) phosphorylated IRE1α (pIRE1α), (**C**) spliced XBP1 (sXBP1), (**D**) CHOP and (**E**) cleaved caspase-12 (c-Casp-12) were determined by Western blotting (WB) in homogenates of lungs collected 20 h after CLP or sham operation. The levels of all five ER stress proteins were elevated in septic WT mice (*P < 0.05 vs. sham WT mice) and were significantly reduced in septic CIRP KO mice (^#^*P* < 0.05 vs. septic WT mice). *The dotted lines on the WB reflect the groups shown in the histogram below. Histograms show mean densitometric analysis of bands normalized for β-actin and relative to expression values in sham WT mice. Data are expressed as last square mean* ± *SEM. Sample sizes: sham WT* = *4, CLP WT* = *6 sham CIRP KO* = *4, CLP CIRP KO* = *6*.

ER stress is a known inducer of apoptosis^10^. To determine whether CIRP activates ER stress in ALI, we examined the protein expression levels of key ER stress markers in the lung at 20 h after CLP. Compared with the sham group, the lungs of WT mice with sepsis-associated ALI had significantly increased levels of the chaperone protein BiP (1.8-fold), phosphorylated (activated) ER stress sensor pIRE1α (1.9-fold), spliced (activated) transcription factor sXBP1 (1.7-fold), and pro-apoptotic components CHOP (1.6-fold) and cleaved (activated) caspase-12 (1.3-fold) (Fig. 3A–E). In CIRP KO mice, however, these markers barely increased in the lungs of septic mice compared with the sham group (Fig. 3A–E). The baseline level of ER stress markers in sham CIRP KO mice was similar to that of sham WT mice. These results demonstrate that CIRP induces ER stress in sepsis-associated ALI.

### CIRP mediates CHOP expression in pulmonary arteriolar endothelial cells in sepsis-associated ALI

**Figure 4:**
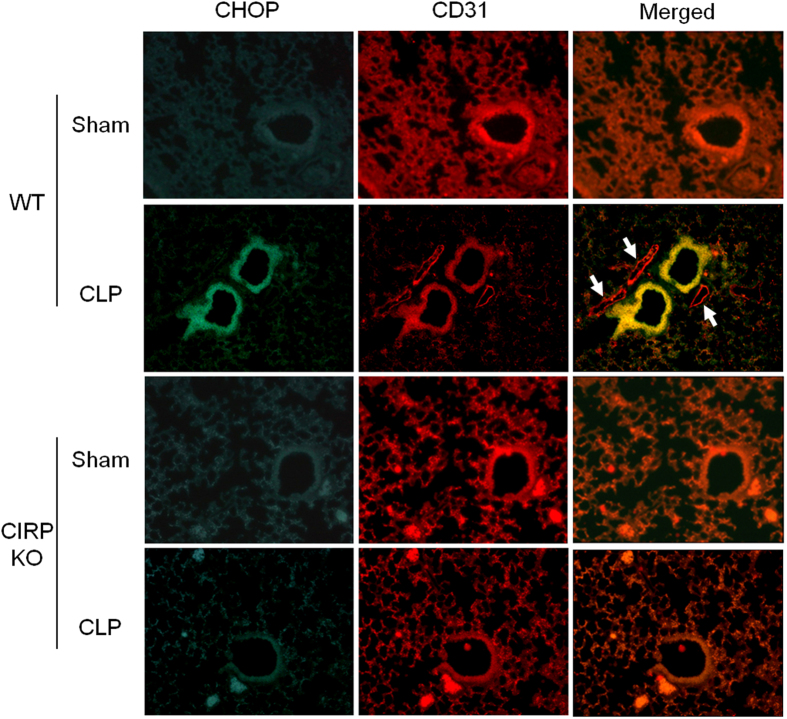
CIRP induces CHOP expression in pulmonary arteriolar and microvascular endothelial cells. The expression of CHOP (green) and CD31 (red) was determined by immunofluorescence in lungs collected 20 h after CLP or sham operation. High expression of CHOP was detected in pulmonary arteriolar endothelial cells of septic WT mice, but not in the other groups. CHOP was highly expressed in arteriolar endothelial cells (merged, yellow), and to a lesser extent in microvascular endothelial cells (merged, orange). CHOP was not detected in venular endothelial cells of septic WT mice (merged, white arrows). *Representative sections; original magnification, 200X*.

To determine more precisely which cells undergo ER stress during ALI, we examined lungs collected at 20 h after CLP or sham operation for CHOP and CD31 expression by immunofluorescence. CD31 is a surface marker of endothelial cells (EC) that is shed upon EC activation by pro-inflammatory cytokines^31^,^32^,^33^,^34^. As expected, capillary expression of CD31 was decreased in the lungs of WT mice with CLP and, to a lesser degree, in CIRP KO mice with CLP. CHOP was markedly upregulated in the lungs of septic WT mice compared to sham WT mice (Fig. 4). CHOP was strongly expressed by pulmonary arteriolar endothelial cells and, to a lesser extent, microvascular endothelial cells, as determined by co-localization with CD31. CHOP expression was not detected in venular endothelial cells. CHOP expression was also not well detected in sham or septic CIRP KO mice (Fig. 4). These observations indicate that, in sepsis-associated ALI, CIRP leads to the expression of CHOP in endothelial cells, and that CHOP is predominantly induced in the pulmonary arterioles.

### CIRP increases inflammation in sepsis-associated ALI

**Figure 5:**
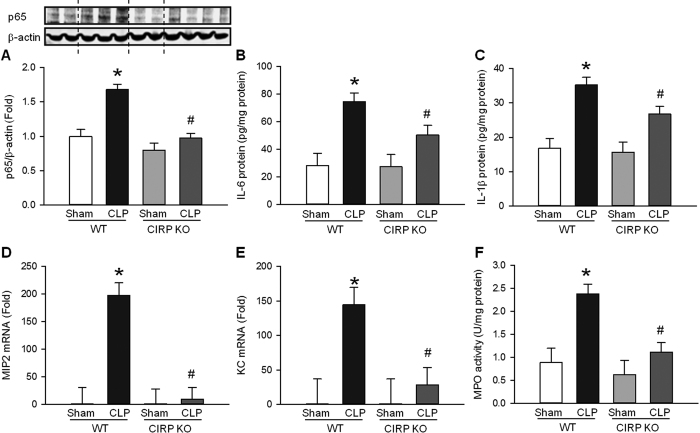
CIRP increases NF-κB, cytokine, and chemokine expression, and neutrophil infiltration in ALI. (**A**) Lung levels of NF-κB p65 (WB) were elevated in septic WT mice (*P < 0.05 vs. sham WT mice) and were significantly reduced in septic CIRP KO mice (^#^*P* < 0.05 vs. septic WT mice). At 20 h after CLP, the lung protein expression of (**B**) IL-6 and (**C**) IL-1β were measured by ELISA, the lung mRNA expression of (**D**) MIP-2 and (**E**) KC were measured by real time-PCR, and (**F**) lung MPO activity was measured by colorimetry. The lung expression of proinflammatory cytokines and chemokines, as well as neutrophil infiltration as measured by MPO activity, were elevated in septic WT mice (*P < 0.05 vs. sham WT mice) and were significantly reduced in septic CIRP KO mice (^#^*P* < 0.05 vs. septic WT mice). *The dotted lines on the WB reflect the groups shown in the histogram below. Data are expressed as last square mean* ± *SEM. Sample sizes: sham WT* = *2–4, CLP WT* = *2–8, sham CIRP KO* = *2–4, CLP CIRP KO* = *4–8*.

ER stress is a key inducer of proinflammatory cytokine and chemokine expression^26^,^35^,^36^. Therefore, we investigated the contribution of CIRP to inflammation in the lungs of mice subjected to CLP or sham operation. At 20 h after CLP, p65 (an NF-κB subunit) was 1.7-fold higher in the lungs of septic WT mice compared with sham WT mice (Fig. 5A). The levels of p65 in septic CIRP KO mice, however, were not elevated when compared with sham CIRP KO mice (Fig. 5A). Next, we measured the levels of proinflammatory cytokines and chemokines known to play a significant role in sepsis-mediated ALI^37^. IL-6 and IL-1β levels were 2.6- and 2.1-fold higher, respectively, in the lungs of septic WT mice than in sham WT mice, and were reduced by 49.8% and 40.2%, respectively, in septic CIRP KO mice compared to septic WT mice (Fig. 5B–C). Similarly, mRNA levels of MIP-2 and KC were 198.0- and 144.5-fold higher, respectively, in septic WT mice compared to sham WT mice. In septic CIRP KO mice, however, mRNA levels of MIP-2 and KC were reduced by 95.9% and 81.1%, respectively, compared to septic WT mice (Fig. 5D–E). MIP-2 and KC are potent neutrophil chemoattractants. Therefore, we also measured myeloperoxidase (MPO) activity, an indicator of the presence of activated neutrophils. MPO activity was 2.7-fold higher in septic WT mice than in sham WT mice (Fig. 5F). MPO activity in septic CIRP KO mice, however, was reduced by 67.3% compared to septic WT mice, indicating decreased neutrophil infiltration in the lungs of CIRP KO mice. Taken together, these results suggest that, similarly to ER stress, CIRP promotes NF-κB activation, inducing the expression and release of proinflammatory cytokines and chemokines and the influx of neutrophils, all of which are key elements in the pathobiology of sepsis-associated ALI.

### CIRP increases oxidative stress and the expression of inducible nitric oxide synthase (iNOS) in sepsis-associated ALI

**Figure 6:**
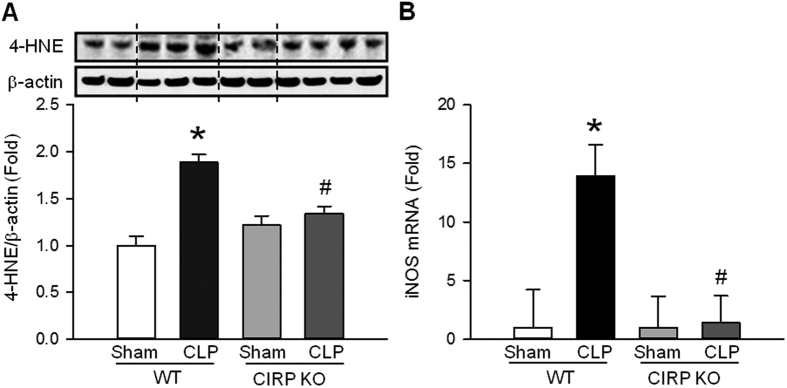
CIRP increases lipid peroxidation and iNOS in ALI. At 20 h after CLP, the lung levels of (**A**) 4-HNE (WB) and (**B**) iNOS (RT-PCR) were elevated in septic WT mice (*P < 0.05 vs. sham WT mice) and were significantly reduced in septic CIRP KO mice (^#^*P* < 0.05 vs. septic WT mice). *The dotted lines on the WB reflect the groups shown in the histogram below. Histograms show data normalized for β-actin and relative to expression values in sham WT mice. Data are expressed as last square mean* ± *SEM. Sample sizes: 4-HNE, sham WT* = *4, CLP WT* = *6, sham CIRP KO* = *4, CLP CIRP KO* = *7; iNOS, WT* = *2, CLP WT* = *3, sham CIRP KO* = *3, CLP CIRP KO* = *4*.

In response to ER stress, there is an increase in the production of reactive oxygen species (ROS)^38^. ROS can cause lipid peroxidation and result in tissue damage^39^. CIRP has also been associated with increased tissue levels of ROS^40^. To determine whether CIRP promotes oxidative stress during ALI, we quantified 4-hydroxy-2-nonenal (4-HNE), a marker of lipid peroxidation, in the lungs of mice subjected to CLP or sham operation. At 20 h after CLP, the levels of 4-HNE were 1.9-fold higher in septic WT mice than in sham WT mice (Fig. 6A). The levels of 4-HNE in septic CIRP KO mice, however, were not elevated when compared to sham CIRP KO mice (Fig. 6A). The ER stress-induced production of ROS is mediated by iNOS^41^. Therefore, we quantified iNOS mRNA in the lung tissues of WT and CIRP KO mice collected 20 h after CLP or sham operation and found that it was 13.7-fold higher in septic WT mice compared with sham WT mice (Fig. 6B). Expression of iNOS mRNA in septic CIRP KO mice, however, was not increased and was similar to that of sham CIRP KO mice (Fig. 6B). Sham CIRP KO and sham WT mice had similar baseline levels of 4-HNE and iNOS. These results indicate that CIRP is an important contributor to the production of ROS during ALI, possibly via induction of iNOS following ER stress.

### CIRP induces ER stress via TLR4

**Figure 7:**
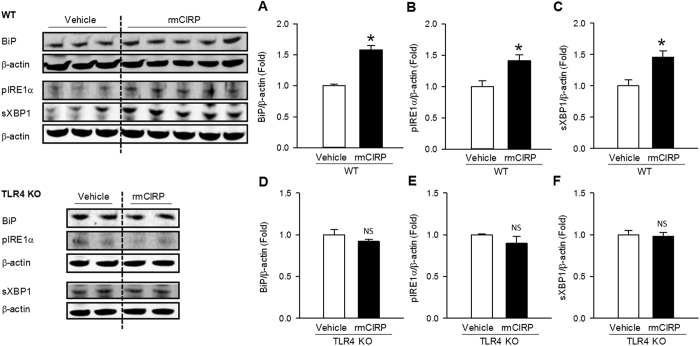
CIRP-induced ER stress is TLR4-dependent. Lung levels of (**A**) BiP, (**B**) pIRE1α, and (**C**) sXBP1 proteins were significantly elevated in healthy WT mice treated with rmCIRP (*P < 0.05), confirming that CIRP induces ER stress. Lung levels of (**D**) BiP, (**E**) pIRE1α, and (**F**) sXBP1 proteins, however, were not increased in healthy TLR4 KO mice treated with rmCIRP (NS, not significant), indicating that ER stress induction by CIRP requires TLR4. *Western blotting of homogenates from lungs collected 20 h after rmCIRP intravenous administration. The dotted lines on the WB reflect the groups shown in the histogram below. Data are expressed as mean* ± *SEM. Sample sizes: WT, vehicle* = *3, rmCIRP* = *5; TLR4 KO, vehicle* = *2, rmCIRP* = *2*.

We have previously shown that CIRP aggravates sepsis via its binding to the TLR4-MD2 receptor complex^7^. To determine how CIRP regulates ER stress, we administered recombinant murine (rm) CIRP or vehicle (phosphate-buffered saline solution) intravenously to healthy WT and TLR4 KO mice. At 20 h after injection, we collected the lungs and measured protein levels of BiP, pIRE1α, and sXBP1. Compared to vehicle, administration of rmCIRP resulted in a statistically significant induction of BiP (1.6-fold), pIRE1α (1.4-fold), and sXBP1 (1.5-fold) in the lungs of WT mice (Fig. 7A–C). Administration of rmCIRP to TLR4 KO mice, however, failed to induce BiP, pIRE1α, or sXBP1 (Fig. 7D–F). These findings indicate that CIRP is sufficient to induce ER stress, and suggest that CIRP regulates ER stress via activation of the TLR4 receptor.

## Discussion

### CIRP is an RNA-binding protein normally present in the cell nucleus

We have previously shown that in sepsis and septic shock, CIRP translocates from the nucleus to the cytosol and is subsequently released into the circulation, where it acts as a DAMP to increase disease severity and mortality^7^. We have also shown that healthy mice injected with CIRP develop lung injury^9^, suggesting that the CIRP released during sepsis contributes to the development of sepsis-associated ALI. In the present study, we discovered a previously unknown link between CIRP and ER stress in the pathogenesis of ALI. Several studies have suggested a role for ER stress in sepsis and ALI pathobiology^10^,^18^,^19^. Here we show that CIRP is critical for the sepsis-induced ER stress response in the lungs of septic mice. CIRP was required for the induction of five key ER stress proteins: the chaperone protein BiP, the phosphorylated (activated) form of the ER stress sensor IRE1α, the spliced (activated) form of transcription factor XBP1, and the pro-apoptotic components CHOP and cleaved (activated) Casp-12. These components of the ER stress response and UPR are fundamental for activation of downstream cascades that ultimately cause the release of proinflammatory mediators and apoptosis^42^,^43^,^44^,^45^. CHOP, for example, contributes to the myocardial dysfunction often present in sepsis by causing cardiomyocyte apoptosis^45^. Additionally, mice with sepsis induced by CLP or lipopolysaccharide (LPS) injection that are deficient in CHOP have lower levels of proinflammatory cytokines, less splenocyte apoptosis, improved bacterial clearance, and increased survival rates^18^. Likewise, septic mice that are deficient in Casp-12 also have decreased levels of proinflammatory cytokines and increased survival after CLP^46^. Although two studies have suggested otherwise^47^,^48^, a large number of studies have implicated Casp-12 in the regulation of ER stress-induced apoptosis. Casp-12 KO mice are resistant to ER stress-induced apoptosis^49^, cultured cortical neurons from Casp-12 KO mice are resistant to amyloid-β neurotoxicity^49^, and pharmacologic modulation of Casp-12 has been shown to regulate ER stress-induced apoptosis^50^,^51^,^52^,^53^.

Among the three ER stress sensors, we focused on the IRE1α pathway because it integrates ER-stress signaling with the inflammatory response, which is the most prominent and clinically relevant feature of ALI. During ER stress, IRE1α associates with the adaptor protein TRAF2 to activate NF-κB and AP-1, two key transcription factors in inflammation and immunity^16^,^17^,^19^,^54^. Upon activation, NF-κB and AP-1 translocate to the nucleus, where they induce the expression of proinflammatory cytokines and chemokines known to promote sepsis-associated ALI^55^. Accordingly, we observed an association between CIRP and expression of the NF-κB protein p65. We also observed that CIRP increased the levels of IL-6, IL-1β, MIP-2, and KC in the lungs of septic mice. MIP-2 and KC are potent chemoattractants that regulate neutrophil migration to the lungs^56^. Indeed, we noticed that CIRP was also associated with increased influx of neutrophils to the lungs. Taken together, these observations suggest that CIRP-induced inflammation and neutrophil influx in ALI may be mediated by ER stress. Our observations are supported by published studies that showed reduced levels of proinflammatory cytokines in septic mice deficient for the CHOP and caspase-12 components of the UPR^18^,^21^,^46^.

We found that CIRP induced ER stress in pulmonary endothelial cells. This finding is in agreement with the fact that, during sepsis-associated ALI, endothelial cells are directly exposed to circulating DAMPs and endotoxin, causing endothelial cell activation leading to increased surface expression of adhesion molecules, dismantling of tight junctions, expression and release of proinflammatory cytokines and chemokines, and acquisition of a prothrombotic phenotype^9^,^57^,^58^. We have recently demonstrated that CIRP alone is sufficient to activate pulmonary endothelial cells, leading to increased surface expression of E-selectin and ICAM-1, assembly and activation of the Nlrp3 inflammasome, and release of IL-1β^9^. Interestingly, ER stress is also known to induce the Nlrp3 inflammasome^13^,^26^. We observed decreased intensity of CD31 straining in the lungs of mice subjected to CLP, suggesting that endothelial cells had been activated and undergone shedding of CD31’s extracellular domain^32^,^33^,^34^.

We observed that sepsis only increased lung apoptosis in the presence of CIRP. Apoptosis is a well-known late result of ER activation, and CHOP is upregulated and induces apoptosis in the lungs of mice with LPS-induced ALI^21^. Furthermore, pulmonary endothelial cell apoptosis is a significant cause of increased vascular permeability contributing to edema, thrombosis, and neutrophil migration in sepsis-associated ALI^30^,^59^,^60^. These observations suggest that ER stress induced by CIRP may aggravate sepsis via apoptosis-associated endothelial cell dysfunction. Pulmonary endothelial cell apoptosis in ALI is dependent on NADPH-oxidase and iNOS activities^30^. In our study, we found a significant association between CIRP, ROS, and iNOS in the lungs of septic mice. CIRP has been reported to induce ROS^40^, and we have shown that CIRP causes activation of the ROS-producing NADPH-oxidase in lung endothelial cells^9^. ER stress is also known to result in increased production of ROS^38^,^61^. ROS-induced peroxidation of biomembrane phospholipids, leading to the formation of 4-HNE, is a relevant and biologically significant consequence of ROS production. Additionally, lipid peroxidation works as an amplification loop to further increase ER stress^62^. Additionally, ER stress-triggered production of ROS is mediated, at least in part, by upregulation of iNOS^41^. In our study, iNOS upregulation required the presence of CIRP. Therefore, it is possible that the CIRP-induced production of ROS in the lungs of septic mice was in fact mediated by CIRP-induced ER stress via iNOS upregulation.

We have shown that injection of exogenous CIRP leads to ALI via CIRP-caused pulmonary endothelial cell activation^9^. As such, we decided to focus our investigation on endothelial cells and observed that CHOP expression was particularly prominent in pulmonary arteriolar endothelial cells, intermediate in microvascular endothelial cells, and not detected in venular endothelial cells. This CHOP expression gradient from the pulmonary artery to the pulmonary vein indicates preferential ER stress activation in the arterial pulmonary circulation and suggests a pre-pulmonary origin for CIRP. Macrophages are a known source of CIRP^7^. As such, it is possible that in sepsis most of the circulating CIRP is released by resident mononuclear phagocytic cells in the two largest solid organs, the liver and spleen.

This is the first time that mice lacking the CIRP protein have been studied for sepsis-associated ALI. We found that CIRP KO mice developed less severe sepsis overall, as measured by serum levels of AST and IL-6, two disease severity biomarkers in sepsis and sepsis-associated ALI^27^,^28^. This finding is consistent with studies that found an association between circulating levels of CIRP and sepsis severity in mice and in humans^7^,^8^, and also with studies that found an association between ER stress activation and CLP- and LPS-induced ALI severity and mortality rates^18^,^21^,^46^. Additionally, decreased levels of IL-6 and other systemic injury biomarkers have also been reported after blockade of CIRP in a number of experimental models of human diseases associated with acute inflammation^7^,^9^,^63^,^64^. We also observed that the presence of the CIRP protein was critical for the development of histological features typical of ALI associated with sepsis in humans and with CLP in mice, namely septal thickening, hyaline membranes, proteinaceous exudates, microthrombi, and neutrophilic infiltrates^65^. CIRP-induced tissue damage has been reported in other models of human disease^63^,^66^,^67^, and is likely the result of the convergence of CIRP’s endothelial-activating, proinflammatory, and pro-apoptotic effects.

We have previously shown that CIRP binds the TLR4-MD2 complex with high affinity^7^. Additionally, TLR4 agonists are known inducers of ER stress^19^,^24^,^25^,^68^,^69^. These two observations suggest that CIRP might cause ER stress via its binding to TLR4. Indeed, we have now shown that CIRP activation of ER stress is TLR4-dependent. Endotoxemia, which is often present in sepsis, can also induce ER stress via activation of TLR4^19^,^25^. Studies show that a functional TLR4 is required for the development of ALI secondary to endotoxemia^70^, hemorrhagic shock^71^,^72^, burns^73^, and paraquat poisoning^74^. Since CIRP is one of TLR4’s known agonists, it is possible that CIRP also plays a role in the development of ALI after these insults.

**Figure 8:**
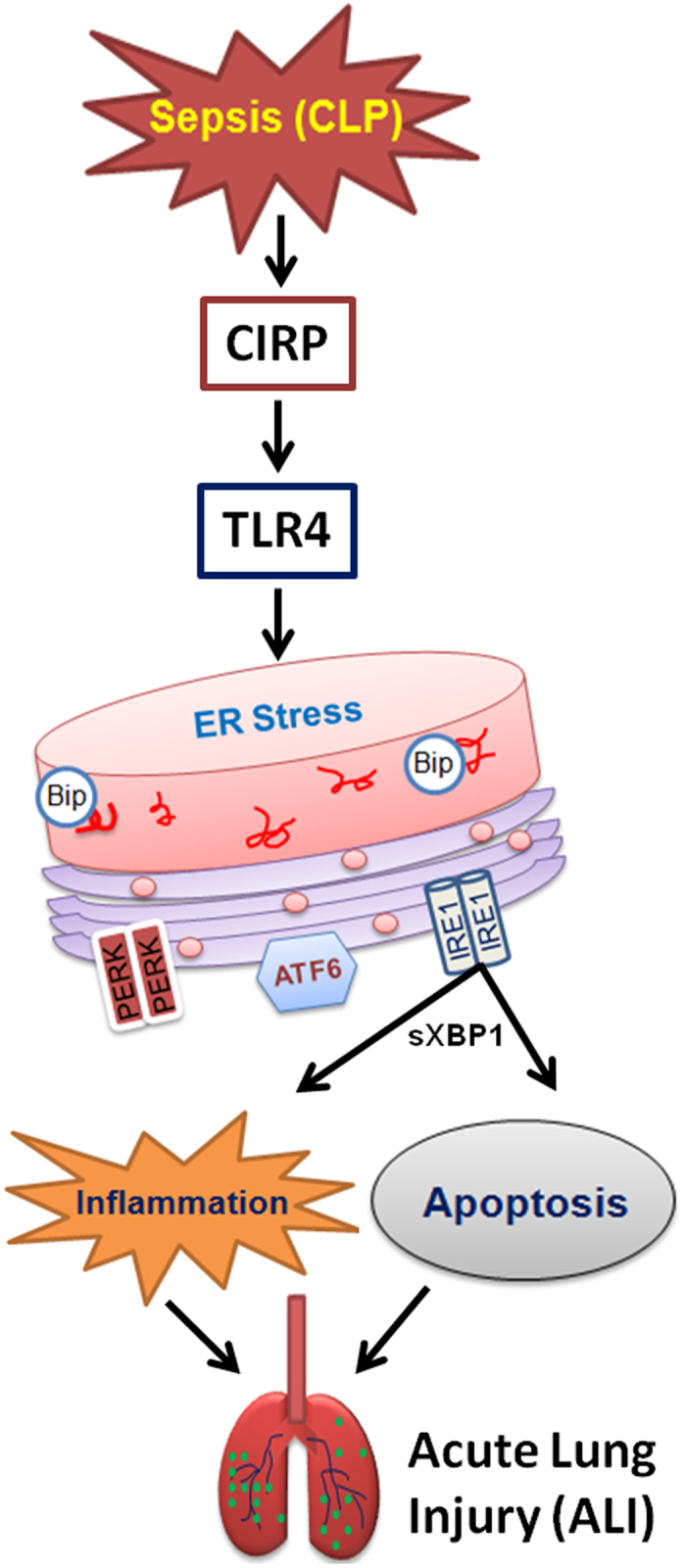
Proposed sequence of events in ALI pathogenesis. During sepsis (CLP), CIRP is released and interacts with TLR4. The CIRP-TLR4 interaction leads to activation of ER stress, causing inflammation and apoptosis, ultimately resulting in acute lung injury (ALI).

In conclusion, we discovered a novel pathway linking CIRP to the induction of ER stress in the development of sepsis-associated ALI (Fig. 8). This new pathway is TLR4-dependent and involves local induction of proinflammatory cytokines and chemokines, neutrophil influx, and apoptosis. Future studies should consider agents targeting CIRP as a potential new therapeutic strategy to treat patients with sepsis.

## Methods

### Animals

Male 8- to 12-week-old wild type (WT, C57BL/6), CIRP KO (*Cirbp*^−/−^), and TLR4 KO (*Tlr4*^−/−^) mice were used in the experiments. CIRP KO mice^75^,^76^ and TLR4 KO mice^77^ both have a C57BL/6 background and were kind gifts from Dr. Jun Fujita (Kyoto University, Japan) and Dr. Huan Yang (FIMR), respectively. Mice were housed in temperature-controlled rooms with 12-h light cycles and fed a standard mouse diet. All experiments involving live animals were performed in accordance with the National Institutes of Health guidelines for use of experimental animals, and approved by the FIMR Institutional Animal Care and Use Committee.

### Animal model of sepsis

Mice were anesthetized with isoflurane and placed in the supine position. The ventral abdomen was shaved and cleaned with 10% povidone-iodine wash and CLP was performed as described previously^7^. Briefly, a 1-cm midline incision was performed. The cecum was exposed, ligated just distal to the ileocecal valve to avoid intestinal obstruction, and punctured twice with a 22-gauge needle. A small amount of cecal content was then expressed from the perforated sites and the ligated cecum was returned to the peritoneal cavity. The laparotomy wound was suture-closed in layers. Sham operated animals underwent the same procedure with the exception that the cecum was neither ligated nor punctured. Immediately after surgery, animals received a subcutaneous injection of 1 ml normal saline. At 20 h after CLP, mice were euthanized for collection of blood and lungs.

### Measurements of serum enzymes and cytokines

Whole-blood samples were centrifuged at 4,000 g for 10 min to collect serum. The activity of aspartate aminotransferase (AST) was determined using a commercial assay kit (Pointe Scientific, Lincoln Park, MI). Serum and tissue levels of interleukin 1β (IL-1β) and interleukin 6 (IL-6) were determined using mouse-specific ELISA kits (BD Biosciences, San Diego, CA). All assays were carried out according to manufacturer’s instructions.

### Histological evaluation of lung injury and TUNEL staining

Upper and lower lobe lung samples were collected 20 h after CLP, fixed in 10% formalin, embedded in paraffin, microsectioned at 4 μm, and stained with hematoxylin-eosin. Lung injury score was then assessed in a blinded fashion using a semi-quantitative light microscopy evaluation as previously described^28^. For terminal deoxynucleotidyl transferase dUTP nick-end labeling (TUNEL) staining, fluorescence staining was performed using a commercially available *in situ* Cell Death Detection Kit (Roche Diagnostics, Indianapolis, IN). The assay was conducted according to the manufacturer’s instructions. Results are expressed as the average number of TUNEL-positive staining cells per 200X magnification field.

### Western blotting

Lungs from each group of mice were homogenized in 500 μl lysis buffer (10 mM Tris-HCL pH 7.5, 120 mM NaCl, 1% NP-40, 1% sodium deoxycholate, and 1% Triton X-100) containing protease and phosphatase inhibitor cocktails (Roche) using a sonicator on ice. Samples were centrifuged at 14,000 *g* for 20 min at 4 °C, and the supernatants were collected. Sample protein concentrations were measured using the BCA protein assay kit (Pierce Biotechnology, Rockford, IL), and 65-μg protein samples were separated by electrophoresis on 4–12% gradient Bis-Tris gels and transferred to nitrocellulose membranes. After blocking with 0.1% casein, membranes were incubated with primary antibodies against BiP, pIRE1α, CHOP, cleaved caspase-12 (Cell signaling Technology, Danvers, MA), sXBP1, NF-κ p65 (Santa Cruz Biotechnology, Santa Cruz, CA), 4-HNE (Abcam, Cambridge, MA), or β-actin (Sigma-Aldrich, St. Louis, MO). 4-HNE is stable, making its quantification more reliable than direct quantification of ROS

After washing, membranes were incubated with the appropriate fluorescent-conjugated secondary antibodies. Bands were detected using an Odyssey FC Imaging system (LI-COR, Lincoln, NE). Band intensities were quantified with densitometry and represented as fold changes relative to controls. The corrected band intensity data from two Western blots was used to plot each histogram.

### Immunofluorescence confocal microscopy

Fixed lung histological sections were first stained with unconjugated anti-mouse CHOP (clone L63F7) monoclonal antibody (Cell Signaling), then stained with Alexa Fluor^®^ 488-labeled polyclonal anti-mouse IgG (H + L) F(ab’)_2_ antibody fragment (Cell Signaling), and then stained for ECs with phycoerythrin (PE)-labeled anti-mouse CD31 antibody (clone MEC13.3) (Biolegend, San Diego, CA). The stained lung section was imaged by confocal microscope with excitation at 480 nm for green fluorescence (CHOP) and at 540 nm for red fluorescence (CD31).

### Real-time reverse transcription polymerase chain reaction analysis

RNA was extracted from lung tissues using TRIzol (Invitrogen, Carlsbad, CA) and reverse-transcribed into cDNA using murine leukemia virus reverse transcriptase (ThermoFisher Scientific, Waltham, MA). PCR reactions were carried out in 25 μl containing 0.08 μmol of each forward and reverse primer (Table 1), 5 μl cDNA, 6.5 μl H_2_O, and 12.5 μl SYBR Green PCR Master Mix (ThermoFisher Scientific). Amplification was conducted in duplicates using a 7300 real-time thermocycler (Applied Biosystems, Foster City, CA) with the thermal profile of 50 °C for 2 min and 95 °C for 10 min, followed by 45 cycles of 95 °C for 15 s and 60 °C for 1 min. The level of mouse β-actin mRNA was used for normalization. Relative expression of mRNA was determined using the 2^(−ΔΔ*Ct*)^ method. The primer sequences are listed as following:

### Myeloperoxidase activity assay

Lung tissues were sonicated in 50 mM potassium phosphate buffer containing 0.5% hexadecyltrimethylammonium bromide. After centrifugation, the supernatant was diluted in reaction solution containing o-dianisidine hydrochloride and H_2_O_2_. The rate of change in optical density (OD) for 1 min was measured at 460 nm to calculate MPO activity, as described previously^7^.

### Administration of rmCIRP

The expression and purification of rmCIRP has been previously described^7^. Briefly, rmCIRP was expressed in *E. coli*, purified, and validated by SDS-PAGE, Western blotting, and liquid chromatography tandem mass spectrometry. rmCIRP (5 mg/kg BW) or vehicle (phosphate-buffered saline solution, PBS) was administered to healthy WT and TLR4 KO mice through the jugular vein. Lungs tissues were harvested at 20 h post-injection for the measurement of ER stress markers.

### Statistical analysis

Results are reported as least square mean ± SEM. Sample sizes assumed a two-tailed statistical significance of 0.05 or less and a power of 0.8. Sample sizes for sham groups included a reduced number of animals because measurements in these groups are significantly more homogeneous. Specific measurements with smaller sample sizes were sometimes used because of limited amount of serum/tissue left, and reflect random sampling rather than exclusion. Analyses of multiple groups were carried out using two-way ANOVA with multiple comparisons using Dunnett’s method with WT sham as the control group. Comparisons between two groups were carried out using two-tailed Student’s t-test or the Mann-Whitney test, depending on the distribution.

## Additional Information

**How to cite this article**: Khan, M. M. *et al*. Cold-inducible RNA-binding protein (CIRP) causes sepsis-associated acute lung injury via induction of endoplasmic reticulum stress. *Sci. Rep.* **7**, 41363; doi: 10.1038/srep41363 (2017).

**Publisher's note:** Springer Nature remains neutral with regard to jurisdictional claims in published maps and institutional affiliations.

## Figures and Tables

**Table 1:** Primer sequences used for cDNA amplification.

Protein	Gene	RefSeq	Forward primer	Reverse primer
iNOS	*Nos2*	NM_010927	GGCAAACCCAAGGTCTACGTT	GAGCACGCTGAGTACCTCATTG
IL-6	*Il6*	NM_031168	CCGGAGAGGAGACTTCACAG	CAGAATTGCCATTGCACAAC
MIP-2	*Cxcl2*	NM_009140.2	CATCCAGAGCTTGAGTGTGA	CTTTGGTTCTTCCGTTGAGG
KC	*Cxcl1*	NM_008176	GCTGGGATTCACCTCAAGAA	ACAGGTGCCATCAGAGCAGT
